# SPIM-Flow: An Integrated Light Sheet and Microfluidics Platform for Hydrodynamic Studies of *Hydra*

**DOI:** 10.3390/biology12010116

**Published:** 2023-01-11

**Authors:** Per Niklas Hedde, Brian T. Le, Erika L. Gomez, Leora Duong, Robert E. Steele, Siavash Ahrar

**Affiliations:** 1 Beckman Laser Institute and Medical Clinic, University of California Irvine, Irvine, CA 92612, USA; 2 Department of Biomedical Engineering, CSU Long Beach, Long Beach, CA 90840, USA; 3 Department of Molecular Biology and Biochemistry, University of California Irvine, Irvine, CA 92697, USA; 4 Department of Biological Chemistry, University of California Irvine, Irvine, CA 92697, USA; 5 Department of Physics and Astronomy, University of California Irvine, Irvine, CA 92697, USA

**Keywords:** light sheet, microfluidics, *Hydra*, hydrodynamics

## Abstract

**Simple Summary:**

This work demonstrates an inexpensive light sheet microscope that can be readily integrated with modern microfluidics. The integration between these two technologies is critical for investigating the hydrodynamics of aquatic organisms. As a case study, we examined the hydrodynamics of *Hydra* in millimeter-sized chambers. The platform was used to study *Hydra*’s response to flow and detachment from channel surfaces. Broader access to light sheet microscopy, particularly integrated with fluidics, could be critical for providing exciting opportunities to investigate the hydrodynamics of freely moving model organisms.

**Abstract:**

Selective plane illumination microscopy (SPIM), or light sheet microscopy, is a powerful imaging approach. However, access to and interfacing microscopes with microfluidics have remained challenging. Complex interfacing with microfluidics has limited the SPIM’s utility for studying the hydrodynamics of freely moving multicellular organisms. We developed SPIM-Flow, an inexpensive light sheet platform that enables easy integration with microfluidics. We used SPIM-Flow to investigate the hydrodynamics of a freely moving *Hydra* polyp via particle tracking in millimeter-sized chambers. Initial experiments across multiple animals, feeding on a chip (*Artemia franciscana* nauplii used as food), and baseline behaviors (tentacle swaying, elongation, and bending) indicated the organisms’ health inside the system. Fluidics were used to investigate *Hydra*’s response to flow. The results suggested that the animals responded to an established flow by bending and swaying their tentacles in the flow direction. Finally, using SPIM-Flow in a proof-of-concept experiment, the shear stress required to detach an animal from a surface was demonstrated. Our results demonstrated SPIM-Flow’s utility for investigating the hydrodynamics of freely moving animals.

## 1. Introduction

Need for SPIM: Selective plane illumination microscopy (SPIM), or light sheet microscopy, is a powerful approach with high spatio-temporal resolution for biological samples imaging [1]. Typically, the excitation arm (i.e., the light sheet path) of a microscope is arranged at a 90-degree angle relative to the detection arm (i.e., the camera path) [2]. In an observation plane, an excitation light is restricted to a thin illumination plane via cylindrical optics or other beam shaping methods. This one-dimensional confinement provides true optical sectioning, and it limits the phototoxicity and photobleaching of the sample, thus enabling long-term imaging [1]. Many SPIM platforms have been demonstrated [3]. Examples include open-source approaches [4,5], systems that use a single objective [6], and even systems that use mobile phones for imaging [7]. SPIM has enabled biological discoveries across sub-cellular imaging [8,9], developmental studies (across vertebrates, invertebrates, and plant model systems) [10,11,12], and optically cleared tissues (e.g., the entire brains of small animals) [13]. While SPIM provides a powerful approach for long-term imaging, samples are often housed in static conditions. Significant modifications to fluidics are required when fluidics are used to accommodate light sheet microscopy. Thus, interfacing SPIM with conventional microfluidics could further enhance the approach’s utility by allowing the dynamic manipulation of a sample’s microenvironment and broadening the use of the technique [14].

Integrating SPIM with fluidics: Various strategies have been explored to integrate fluidics with light sheet microscopy [15,16]. Such integration has enabled cytometry platforms [17,18,19,20], the generation and visualization of droplets [21], and visualizing *C. elegans* and *Drosophila* embryos [22,23]. For example, a recent and exciting study by Vanwalleghem et al. demonstrated the strength of integration for studying larval zebrafish brain-wide activity while using flow as a stimulus [24]. In previous work, we explored using a light sheet configuration (based on an inverted epifluorescence microscope) which could accommodate chambers with flow [25]. Despite this progress, integrating SPIM with microfluidics has remained difficult. For example, the integration requires modifications to the microscope or significant modifications to the microchambers [15]. Additionally, the total cost (for example, for approaches that augment high-end research microscopes with light sheet capabilities) and the complexity of establishing light sheet systems have precluded their wide adoption across the microfluidic and lab-on-a-chip community. In particular, light sheet illumination can be of great value for microfluidic applications which require particle image tracking and velocimetry. To address this gap, we developed SPIM-Flow, a simple and inexpensive system that readily integrates light sheet microscopy with fluidics. As a proof-of-concept study, we used SPIM-Flow to investigate the hydrodynamics of a freely moving *Hydra* polyps in a millimeter-sized chamber (4 mm wide and 1.6 mm in height). Specifically, instead of utilizing the 3D imaging capabilities of the approach, SPIM-Flow was used to side-illuminate the freely moving organisms, and micron-sized tracer particles were used to visualize the hydrodynamics.

*Hydra* and microfluidics—an approach to investigate hydrodynamics: *Hydra* is a freshwater cnidarian known for its remarkable regenerative abilities [26]. Its transparent, tube-shaped body is divided into three regions: head, body column, and foot. The head includes the tentacles and hypostome—a dome-like structure containing the mouth opening at its apex. *Hydra* uses its foot (i.e., a basal disk) to attach to surfaces. The shape and movement of the body are controlled via a hydrostatic skeleton where fluid pressure transmits forces [26,27]. With these characteristics, *Hydra* is an excellent system to use to investigate biomechanics and hydrodynamics. A recent study, for example, using imaging and machine learning, showed that the behavioral repertoire of *Hydra* could be divided into six components (i.e., elongation, tentacle sway, body sway, bending, contraction, and somersaulting) [28]. In other studies, it was shown that *Hydra* must tear a hole through its epithelial tissue to open its mouth [29,30]. However, these investigations were conducted under static conditions. Using fluid chambers would allow one to expand upon the current studies by modulating the microenvironments’ physical (e.g., flow) or chemical (e.g., transitory drug delivery) compositions. Additionally, previous applications of light sheet microscopy to study *Hydra* have used static chambers [31,32].

In another recent study, Badhiwala et al. developed three microfluidic systems to study *Hydra* [33]. These included a chamber to constrain the body column and enable electrophysiology, a perfusion chamber for constrained locomotion, and a quasi-2D plane behavioral chamber (200–600 µm in height). More recently, the investigators used a double-layer microfluidic system to mechanically stimulate *Hydra* (via pneumatic valves) and measure neuronal responses via calcium imaging [34]. Microfluidic platforms enabled neuronal studies that required animal immobilization or small chambers. However, these restrictions limit an organism’s movement and may negatively impact its health. In our study, by taking advantage of SPIM-Flow, we sought to investigate *Hydra*’s response to flow without dramatically restricting its movement. Chambers with millimeter-sized dimensions (4 mm wide and 1.6 mm tall) were constructed. Next, we utilized the integration of microfluidics to modulate the hydrodynamic environment, for example, by introducing flow or prey. We used the light sheet system to visualize the hydrodynamics of the animal’s movement. Our studies suggested that *Hydra* typically remained in an elongated or swaying state inside the chambers without flow. *Hydra* responds to flow initiation via contraction or tentacle swaying. Moreover, *Hydra* typically reorients by body bending (head and tentacles) in the flow direction. Finally, utilizing the system, the shear stress (at the surface of the chamber) required to detach *Hydra* from devices was measured.

## 2. Materials and Methods

**Figure 1:**
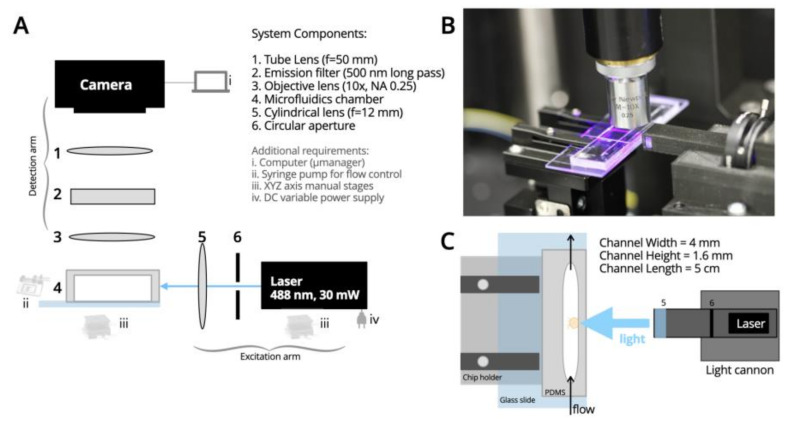
SPIM-Flow system diagram. (**A**) Simple and inexpensive SPIM designed for compatibility with fluidics. (**B**) A photograph of the excitation arm (i.e., the light cannon), microfluidic chamber held on the 3D printed stage, and the objective lens collecting fluorescent signals from the chamber. (**C**) Diagram of the PDMS chamber with its critical dimensions, the stage, and the light cannon. Steps were taken to remove any roughness from the side of the chamber that accepted the light and to remove any rough edges that could create imaging artifacts.

Optical System: The optical system of SPIM-Flow is composed of excitation and detection arms (Figure 1). To create the light sheet, similar to our prior efforts [8], the central part of the elliptical beam of a laser diode (wavelength 488 nm and 30 mW, variant dot/point and TTL output, LambdaWave Turquoise Laser) was cut with an iris (SM1D12, Thorlabs, Newton, NJ, USA). Then, a cylindrical lens (f = 12.7 mm, LJ1942L1-A, Thorlabs) was used to focus and form a sheet of light. These components were housed inside a custom-designed, 3D-printed enclosure. A cannon-like design (i.e., a light-cannon) was developed to bring the cylindrical lens close to the fluidic chip. The 3D-printed enclosure containing the optical components for excitation was then mounted on an XYZ micromanipulator. A light sheet of 7 µm thickness was created utilizing these components (see Supplementary Information for analysis). The excitation arm was mounted on an optical breadboard at a 90-degree angle relative to the detection arm. The detection arm included a custom-designed 3D-printed chip holder to bring the fluidic chip close to the light-cannon. Fluorescent signals were collected perpendicular to the excitation with an objective lens (10×, NA 0.25, Newport Spectra-Physics, Milpitas, CA, USA), followed by an emission filter (500 nm long pass, ET500lp, Chroma, Bellows Falls, VT, USA) and a tube lens (f = 50 mm, AC254-050-A, Thorlabs) for imaging with a CMOS camera (20 MP, FLIR Blackfly S-U3-200S6C-C, Teledyne FLIR, Goleta, CA, USA). A SPIM-Flow system-level diagram is provided (Figure 1A). The system was housed on a small optical breadboard (300 mm × 300 mm) and was inside an opaque enclosure to shield the setup from external light. Micro-Manager software [35] was used to control the camera. Designs for the 3D printed components are available from a corresponding OSF page. Unlike systems that augment a standard microscope (e.g., using an inverted wide-file standard microscope as the detection system) [36], SPIM-Flow is a standalone and portable instrument.

Microfluidic systems: A desktop CO_2_ laser cutter was used to build the millimeter-sized molds used in the study instead of using the conventional lithographic approaches. This choice was informed by the size of *Hydra* (an average length of 10 mm) and our goal to investigate their unhindered hydrodynamics. To this aim, channels were designed to be 4 mm wide and 5 cm long. Acrylic sheets (1/16th inch, 1.6 mm thickness) were laser-cut (40 W Glowforge desktop systems). Plastic parts were retrieved from the sheet and permanently attached to a flat base (either a plastic part or a glass slide) via an adhesive. The attachment prevented the unwanted PDMS accumulation under the mold during the silicone casting. After the mold fabrication, conventional protocols were used to cast silicone parts from the mold (1:10 linker to the base mass ratio, followed by baking the molds on hotplates at 85 °C until the PDMS was fully cured). Individual PDMS devices were cut from the mold using craft knives, and inlet/outlet holes were introduced using disposable (1 or 2 mm in diameter) biopsy punches.

Next, each PDMS part was plasma-bonded to a microscope glass slide via a Harrick Basic Plasma cleaner. The PDMS parts were placed on the edge of the glass slide (instead of at the center) to minimize the distance between the channel and the light cannon (Figure 1B,C). Additionally, steps were taken to remove any roughness from the outer edges of the devices to minimize potential imaging artifacts (some roughness can be introduced to the outer edges of PDMS parts while cutting them via a craft knife). In a typical epifluorescent illumination, such roughness is of no concern since the path of light is through the device top (typically, the PDMS) and glass bottom. In our system, however, the chamber was illuminated through a PDMS wall and viewed from the device’s top. Additional uncured PDMS was applied to the device’s outer wall using a craft knife after plasma-bonding to remove any roughness. Next, the devices, with the side with fresh PDMS facing up, were left at room temperature for 3–4 h. This pause allowed the PDMS to spread evenly and partially cure. Devices were then backed at 95 °C on hotplates to ensure that the added PDMS was entirely cured. During the early experiments, we also attached coverslips to the device roofs. However, later experiments demonstrated that the coverslip did not significantly improve imaging.

*Hydra* culture: All experiments were carried out using the PT1 transgenic line of *Hydra* vulgaris [37]. PT1 contains two transgenes. One of the transgenes expresses green fluorescent protein under the control of the promoter from the gene encoding the Hym176B neuropeptide. The other transgene expresses the gene for the red fluorescent protein, the DsRed2 gene, under the control of an actin gene promoter. This line expresses green fluorescent neurons and red fluorescent epithelial cells. PT1 was maintained in *Hydra* medium 4.0, which was prepared using house deionized water and that contained 1 mM calcium chloride, 0.33 mM magnesium sulfate, 0.5 mM sodium bicarbonate, and 0.03 mM potassium chloride. The animals were fed once a week with the nauplii of *Artemia franciscana* from San Francisco Bay (Brine Shrimp Direct, Ogden, UT, USA). The *Hydra* cultures were kept in an incubator at 18 °C on a 12 h light/12 h dark cycle.

Loading the *Hydra* in microfluidics: A simple protocol was developed to minimize potential damage and increase the successful loading of the animals. First, a device was half-filled with fresh medium. A glass Pasteur pipette was used to retrieve an animal from a culture tube. The biggest challenge was ensuring *Hydra* adhered to the glass transfer pipette, since *Hydra* can rapidly adhere and remain firmly attached to surfaces. Therefore, it was essential to transfer each *Hydra* quickly. The animals were placed directly inside a device inlet or on top of an inlet with excess medium from the pipette. Next, the liquid was withdrawn with a micropipette (1 mL) or a syringe to draw the animal with the additional liquid into the chamber. Each *Hydra* was positioned toward the chamber’s center for ease of imaging. Red fluorescent beads (1 µm diameter, Fluoro-Max Polymer microspheres, ThermoFisher Scientific, Waltham, MA, USA) were added to the medium to enable flow and hydrodynamic visualizations. After establishing the protocol, the animal loading process had a near-perfect success rate.

After loading an animal, the chamber was placed and secured on the 3D-printed chip holder. *Hydra* medium with beads was delivered to the chamber using an external syringe pump (KDS Legato, 210P series) via 3, 10, or 20 mL syringes. Typical of all microfluidics experiments, care was taken to ensure there were no air bubbles upstream of the device. However, small bubbles did not lead to animal death. For example, early in the studies, air bubbles were accidentally introduced into two chambers. In each case, the animal rapidly responded to the air bubble and the pressure by contracting/bending. The animals were able to survive these exposures.

## 3. Results

### 3.1. Health and Feeding

At the start of the study, to verify the animals’ health in the SPIM-Flow, feeding behaviors were investigated. After loading a *Hydra* onto a chip, multiple prey animals (*Artemia franciscana*) were added to the chamber. First, imaging without the light sheet capabilities was used to investigate the potentially harmful effects of the chamber. Each *Hydra* could successfully capture and eat multiple (two to three) *Artemia nauplii* inside the chamber. Next, feeding behavior was observed while using the SPIM imaging capabilities across multiple independent animals (Supplementary Figure S1 and Supplementary Video S1, though the video and images are from two different animals). After feeding, the *Hydra* ignored the remaining prey in the chamber. The feeding results (across N = 4 different animals) suggested that the fluid chambers and the light sheet did not negatively impact the organisms’ health. Subsequent hydrodynamic experiments typically took 4 to 5 h. However, it was confirmed that the *Hydra* could be kept alive on the same chip for multiple days.

### 3.2. Static Conditions 

After examining the animals’ health, we sought to investigate the behavioral repertoire of the *Hydra* without flow. The animals’ movements were unhindered, given the millimeter-sized dimensions of the fluidic chambers. However, some animals could stretch across the entire 4 mm-width of the chamber. Utilizing the XYZ-stages, light sheet illumination could be positioned based on an animal’s positioning. Previous studies have established that *Hydra* respond to light as a stimulus [38,39,40,41]. Therefore, the experiments were started 5 min after the introduction of the light sheet. Additionally, the light was kept on during most of the recording session. SPIM’s capability to limit the light exposure (here, only to the 7 µm-thick illuminated sheet) prevented the potential of photobleaching or phototoxicity. 

**Figure 2:**
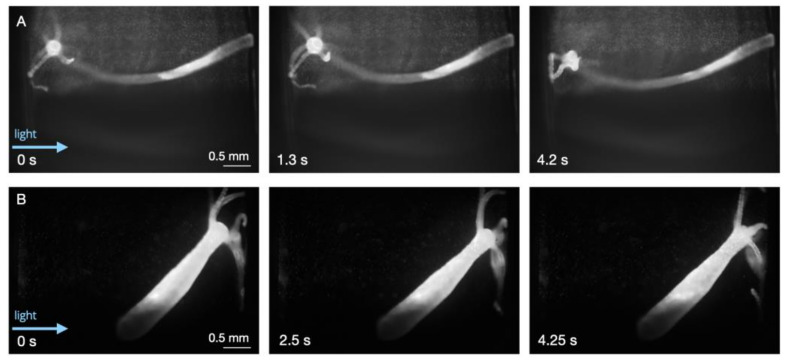
Baseline behavior during static conditions as an indicator of overall health. (**A**) Elongation and tentacle movement. The head (hypostome and tentacle ring) are also visible. (**B**) A different animal in an elongated state inside a chamber. Using SPIM-Flow, GFP-positive neurons could be visualized, depending on the orientation and state of the *Hydra* (excitation 488 nm and emission 500 nm long pass).

**Figure 3:**
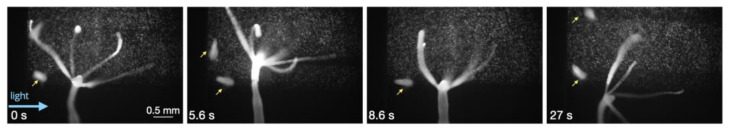
Baseline behaviors (bending and tentacle movement) during static conditions. Yellow arrows point to *Artemia franciscana* nauplii that were ignored by the *Hydra* after capturing 3 nauplii earlier.

Most animals adhered to the surfaces (PDMS walls) closer to the direction of the excitation light (wavelength of 488 nm). Without flow, behaviors ranging from elongation to tentacle movement/swaying were observed (Figure 2 and Figure 3, Supplementary Figure S2, and Supplementary Video S2). Most animals would remain in an elongated or tentacle-swaying state. These observations further supported the assumptions regarding the animals’ overall health and helped demonstrate that various repertoires of the animals’ movements could be captured via the SPIM-Flow.

### 3.3. Response to Flow 

**Figure 4:**
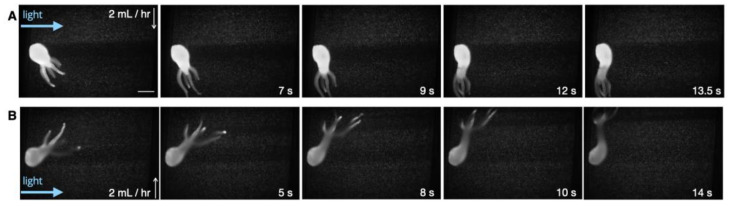
Response to flow. Our results suggested that *Hydra* typically bend and sway their tentacles in the direction of the flow (head aways from the direction of the flow). (**A**) Initiation of the flow and redirection of the animal towards the flow. The white arrow demonstrates the flow direction. The scale bar is 0.5 mm. (**B**) Reversing the flow direction led to the animal redirecting with the new flow direction.

Next, taking advantage of microfluidics, *Hydra*’s response to flow was investigated. *Hydra* live in bodies of freshwater that may experience local flow due to environmental perturbations or currents (in streams and rivers). Therefore, tools to investigate their response to flow could provide valuable insights into the organisms’ biomechanical and hydrodynamic lifestyles. In their study, Badhiwala et al. [33] commented that these animals typically bend in the flow direction. We sought to investigate this observation by utilizing our larger chambers (1.6 mm in height and 4 mm wide). Our results (across four independent animals) suggested that *Hydra* typically respond to flow (2 mL/h) by bending and swaying their tentacles in the flow direction (tentacle-swaying in the direction of the flow (Supplementary Video S3). Reversing the direction of flow also resulted in *Hydra* redirecting accordingly (Figure 4). The results also suggested that the animals could immediately respond to the flow initiation via rapid contraction or tentacle-swaying in the direction of the flow (Supplementary Figure S3).

**Figure 5:**
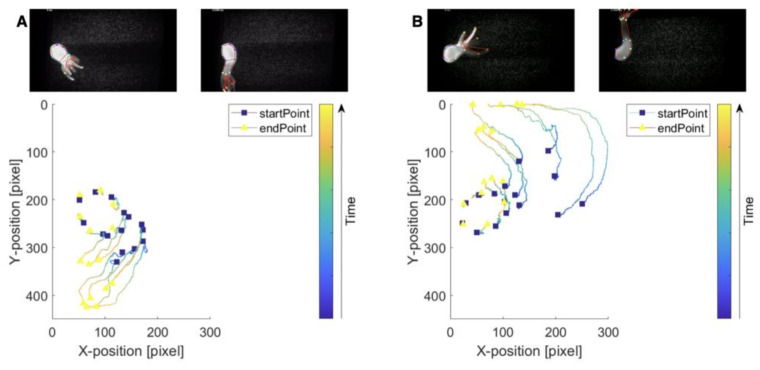
Tracking *Hydra*’s movements in response to flow and pseudo-skeleton visualizations. (**A**) Tracking *Hydra*’s responses to the initiation of flow. Key anatomical features were identified, a pseudo-skeleton was generated, and the movement of features was tracked over space (here, the units are pixels). (**B**) Response to reversing the direction of flow. In each case, tracking and pseudo-skeletons were generated via DeepLabCut. The integration of SPIM-Flow with pose estimation analysis provided pathways for biomechanics investigations. The starting position is highlighted with a blue square. The final position is highlighted with a yellow triangle. The colors indicate the position in time.

Next, two previously described image analysis approaches were used to examine the animals’ responses to flow closely. First, to track the movement of the animals, a markerless pose estimation software (i.e., DeepLabCut2.0) was used [42]. To this aim, locations on the body and the tentacles were identified across video recordings. Utilizing DeepLabCut, the movements of these positions were tracked to generate 2D skeletons of the animals (tentacle-swaying in the direction of the flow (Supplementary Video S4) and plots detailing the changes in the positions of previously identified locations (Figure 5). In the tracking plots, squares represented the starting position, and the triangle represented the final positions for a given body part. The color map indicates the movement of the marker through time (as demonstrated from the first and last frame of the video). Pose estimation software provided opportunities to analyze the results from the SPIM-Flow system experiments. Next, a previously described flow visualization algorithm (i.e., FlowTrace) was used to better visualize the flow features (primarily due to external flow) from the SPIM-Flow recordings. FlowTrace is a simple algorithm that enables the extraction of flow features from videos [43,44]. Unlike typical pipelines for particle image velocimetry, FlowTrace is not computationally taxing. Here, the algorithm was used a FIJI plugin. Therefore, a rapid analysis could provide critical hydrodynamic insights from the SPIM-Flow recordings. The results for these visualizations are provided in Supplementary Figure S4 and Video S5. The videos include both the original recording and the pathline visualizations. 

### 3.4. Adhesion and Detachment from Surfaces 

Finally, using SPIM-Flow, surface shear stress required to detach a *Hydra* specimen from a glass substrate were measured. Investigating *Hydra*’s ability to adhere to surfaces under aqueous environments is of interest for both biomechanical and hydrodynamics. These organisms use adhesive composites (glue) to adhere to various surfaces [45]. Flow chambers could provide a valuable approach to quantitatively measure the shear stress and visualize the flow profiles required to detach *Hydra*. A previous study [46] used a large laminar-flow chamber (tanks) to estimate the forces required to detach these animals from various surfaces. The results suggested that the animal’s size and the substrate play roles in the force required to detach an animal from a surface. However, tracking the fluid elements via particle tracers was absent from the previous system. In this study, we sought to demonstrate SPIM-Flow’s utility for investigating adhesion/detachment. Our platform could provide fine control over the microenvironment and visualize tracer particles, as well as the animals. A previously fed *Hydra* was used to investigate the animal’s detachment from a glass surface. The volumetric flow rate (*Q*) was progressively increased until the *Hydra* specimen was detached from a surface. The animal was rested for 5 min between testing each condition (10 mL/h, 20 mL/h, and 50 mL/h). In our experiments, the animal detached at 50 mL/h. Similar to previously developed analysis [47], the channel’s shear stress at the glass surface (bottom of the chamber) was calculated via:

(1) $$\sigma_{s}=\frac{6Q\mu }{wh^{2}\;},$$

where *Q* is the volumetric flow rate (mL/h), *µ* is the viscosity of *Hydra*’s medium (estimated as the viscosity of the water at 1.002 mPa.s at 20 degrees Celsius), w is the channel width (4 mm), and h is the channel height (1.6 mm). Given the applied low rates of 10–50 mL/h, the corresponding surface shear stress ranged from approximately 1–10 mPa (1.63–8.15 mPa).

## 4. Discussion 

Access to light sheet imaging and interfacing the conventional systems with fluidics have remained challenging. We developed an inexpensive light sheet microscope designed for ready integration with microfluidics (SPIM-Flow) to address these challenges. SPIM-Flow was made by integrating off-the-shelf optics and 3D-printed enclosures. Therefore, the system required no additional optical instruments for its operation. 

This study used SPIM-Flow to investigate the hydrodynamics of freely moving *Hydra* polyps. In particular, the system was used as a miniaturized particle imaging/tracking instead of utilizing the 3D sectioning of the SPIM. To this aim, light sheet imaging enabled an easy approach for the visualization of 1 µm tracer beads. In comparison, the animals were also imaged inside the same microfluidic systems via a standard fluorescent microscope (Nikon Eclipse Ti-E microscope 10X objective—Supplementary Figure S5). Visualizing the beads and the animal (due to the high signal from the animal and the background volume) was challenging with a standard microscope. Additionally, the standard system would illuminate the entire chamber volume throughout the recording session, which could lead to phototoxicity. Our experiments (feeding and baseline behaviors) supported the assumption that the platform did not impact the animals’ health. Feeding on a chip suggested the possibility for the long-term culture and investigation of *Hydra* inside the flow chambers. Next, microfluidics was used to investigate *Hydra’s* response to flow. The results across multiple animals (N = 4) suggested *Hydra* responds to flow by bending in the flow direction (i.e., an animal’s head is facing away from the direction and its tentacles are elongated in the direction of the flow (see Figure 4). Reversing the direction of flow led to animals’ reorientation. Next, SPIM-Flow’s compatibility with two image analysis approaches was demonstrated. In the first analysis, motion tracking software (DeepLabCut) was used to track the location of anatomical landmarks and generate pseudo-skeletons in response to flow. In future studies, landmark tracking could be used to estimate displacements and the speed of movements. Pseudo-skeletons could enable biomechanical studies related to quantifying contractile behaviors. Moreover, DeepLabCut supports automated image annotations with limited user input. In the second image analysis approach, a visualization software (FlowTrace) was used to better demonstrate the tracer beads’ movement, mainly due to the external flow. This study was particularly aimed at investigating the role of flow as environmental stimuli. The combination of SPIM-Flow and FlowTrace in future studies (using higher magnification and larger beads) could enable the investigation of flow patterns generated by *Hydra* alone, without external flows. Finally, in a proof-of-concept experiment, SPIM-Flow was used to visualize and measure the shear forces (50 mL/h, corresponding to surface shear stress of approximately *σ_s_* = 10 mPa) required to detach a *Hydra* specimen from the glass surface of the chamber. Unlike the previous study [46], which used a large laminar-flow tank, SPIM-flow provided a more accessible approach for the fine control and hydrodynamic analysis of bioadhesion. 

SPIM-Flow provided an easy approach for particle tracking/imaging. However, as was apparent in some recordings (see Figure 4), the *Hydra* could obstruct the illumination—due to the side illumination—by casting shadows, creating dark regions in certain portions of the chamber. In future studies, this issue could be addressed by repositioning the light cannon (i.e., illuminating from the opposite side after *Hydra’*s attachment) or, alternatively, by illuminating from both sides. It is also important to acknowledge that the 3D structure of *Hydra* and its fast movements make it difficult to keep the entire animal in focus. This task can be accomplished by restricting the animals, similar to the microfluidic systems previously developed by Badhiwala et al. [33]. However, unlike prior microfluidics with chambers of 200–600 µm in height, the chambers presented in this study did not restrict the movement of the animals (1.6 mm in height). 

## 5. Conclusions

In the future, we plan to use SPIM-Flow to further investigate the hydrodynamics of aquatic organisms (including *Hydra*) by quantifying displacements generated by the animals and systematically exploring the animals’ responses to pulsatile flow. Our preliminary results suggested that pulsatile flow also resulted in an animal stretching toward the flow’s direction. Additionally, light (focused as a sheet) could be used as a stimulus in the systems. In this context, SPIM-Flow could serve as a valuable approach for integrating various environmental stimuli (light, forces/flow, and chemical factors). Microfluidics combined with conventional imaging has provided an excellent platform for studying model systems such as zebrafish, *Drosophila embryos*, and *C. elegans* [48,49]. Other emerging model systems could (and, indeed, have started to) benefit from the needs-driven systematic integration of micro- and milli-fluidics with novel imaging techniques [50,51,52]. Moreover, SPIM-Flow, as a simple and inexpensive platform, can contribute to these studies focusing on the hydrodynamics of freely moving aquatic organisms.

## Data Availability

Data is available upon request.

## Supplementary Materials

The following supporting information can be downloaded at: https://www.mdpi.com/article/10.3390/biology12010116/s1. Supplementary section—analysis of light sheet properties. List of videos and brief descriptions. Supplementary figures. Figure S1: *Hydra* feeding behavior as an indicator of health; Figure S2: *Hydra* baseline behavior in static conditions as an indicator of overall health; Figure S3: Responses to the initiation of flow; Figure S4: Hydrodynamic visualization via FlowTrace; Figure S5: Imaging *Hydra* via a fluorescent microscope; Video S1: *Hydra* feeding behavior (*Artemia franciscana nauplius,* or brine shrimp, used as food); Video S2: *Hydra* tentacle-sway behavior; Video S3: *Hydra* response to flow; Video S4: *Hydra* motion tracking and pseudo-skeleton; Video S5: Hydrodynamic pathline visualizations and sample visualizations (original video and FlowTrace).

## References

[1] Stelzer E.H.K., Strobl F., Chang B.-J., Preusser F., Preibisch S., McDole K., Fiol R. (2021). Light sheet fluorescence microscopy. Nat. Rev. Methods Prim..

[2] Huisken J., Swoger J., Del Bene F., Wittbrodt J., Stelzer E.H.K. (2004). Optical sectioning deep inside live embryos by selective plane illumination microscopy. Science.

[3] Olarte O.E., Andilla J., Gualda E.J., Loza-Alvarez P. (2018). Light-sheet microscopy: A tutorial. Adv. Opt. Photonics.

[4] Voigt F.F., Kirschenbaum D., Platonova E., Campbell R.A.A., Kastli R., Schaettin M., Egolf L., Van der Bourg A., Bethge P., Haenraets K. (2019). The mesospim initiative: Open-source light-sheet microscopes for imaging cleared tissue. Nat. Methods.

[5] Gualda E.J., Pereira H., Vale T., Estrada M.F., Brito C., Moreno N. (2015). Spim-fluid: Open source light-sheet based platform for high-throughput imaging. Biomed. Opt. Express.

[6] Yang B., Lange M., Millett-Sikking A., Zhao X., Bragantini J., VijayKumar S., Kamb M., Gómez-Sjöberg R., Solak A.C., Wang W. (2022). Daxi—High-resolution, large imaging volume and multi-view single-objective light-sheet microscopy. Nat. Methods.

[7] Hedde P.N. (2021). minispim—A miniaturized light-sheet microscope. ACS Sens..

[8] Hedde P.N., Stakic M., Gratton E. (2014). Rapid measurement of molecular transport and interaction inside living cells using single plane illumination. Sci. Rep..

[9] Sapoznik E., Chang B.-J., Huh J., Ju R.J., Azarova E.V., Pohlkamp T., Welf E.S., Broadbent D., Carisey A.F., Stehbens S.J. (2020). A versatile oblique plane microscope for large-scale and high-resolution imaging of subcellular dynamics. Elife.

[10] Lemon W.C., Pulver S.R., Höckendorf B., McDole K., Branson K., Freeman J., Keller P.J. (2015). Whole-central nervous system functional imaging in larval drosophila. Nat. Commun..

[11] Silvestri L., Bria A., Costantini I., Sacconi L., Peng H., Iannello G., Pavone F.S. (2013). Micron-scale resolution optical tomography of entire mouse brains with confocal light sheet microscopy. J. Vis. Exp..

[12] Von Wangenheim D., Hauschild R., Friml J. (2017). Light sheet fluorescence microscopy of plant roots growing on the surface of a gel. J. Vis. Exp..

[13] Ueda H.R., Dodt H.-U., Osten P., Economo M., Chandrashekar J., Keller P. (2020). Whole-brain profiling of cells and circuits in mammals by tissue clearing and light-sheet microscopy. Neuron.

[14] Zhou P., He H., Ma H., Wang S., Hu S. (2022). A review of optical imaging technologies for microfluidics. Micromachines.

[15] Albert-Smet I., Marcos-Vidal A., Vaquero J.J., Desco M., Muñoz-Barrutia A., Ripoll J. (2019). Applications of light-sheet microscopy in microdevices. Front. Neuroanat..

[16] Paiè P., Bragheri F., Bassi A., Osellame R. (2016). Selective plane illumination microscopy on a chip. Lab A Chip.

[17] Regmi R., Mohan K., Mondal P.P. (2013). Light sheet based imaging flow cytometry on a microfluidic platform. Microsc. Res. Tech..

[18] Lin M., Liu Q., Liu C., Qiao X., Shao C., Su X. (2018). Label-free light-sheet microfluidic cytometry for the automatic identification of senescent cells. Biomed. Opt. Express.

[19] Fan Y.-J., Hsieh H.-Y., Tsai S.-F., Wu C.-H., Lee C.-M., Liu Y.-T., Lu C.-H., Chang S.-W., Chen B.-C. (2021). Microfluidic channel integrated with a lattice lightsheet microscopic system for continuous cell imaging. Lab A Chip.

[20] Sala F., Castriotta M., Paiè P., Farina A., D’Annunzio S., Zippo A., Osellame R., Bragheri F., Bassi A. (2020). High-throughput 3d imaging of single cells with light-sheet fluorescence microscopy on chip. Biomed. Opt. Express..

[21] Jiang H., Zhu T., Zhang H., Nie J., Guan Z., Ho C.-M., Liu S., Fei P. (2017). Droplet-based light-sheet fluorescence microscopy for high-throughput sample preparation, 3-d imaging and quantitative analysis on a chip. Lab A Chip.

[22] Behrouzi M., Rahimpouresfahani F., Youssef K., Rezai P., Tabatabaei N. Optofluidic device for light-sheet fluorescence microscopy of c. elegans with a conventional wide-field microscope. Proceedings of the Imaging, Manipulation, and Analysis of Biomolecules, Cells, and Tissues XX.

[23] Memeo R., Paiè P., Sala F., Castriotta M., Guercio C., Vaccari T., Osellame R., Bassi A., Bragheri F. (2021). Automatic imaging of drosophila embryos with light sheet fluorescence microscopy on chip. J. Biophotonics.

[24] Vanwalleghem G., Schuster K., Taylor M.A., Favre-Bulle I.A., Scott E.K. (2020). Brain-wide mapping of water flow perception in zebrafish. J. Neurosci..

[25] Hedde P.N., Malacrida L., Ahrar S., Siryaporn A., Gratton E. (2017). sidespim–selective plane illumination based on a conventional inverted microscope. Biomed. Opt. Express.

[26] Vogg M.C., Galliot B., Tsiairis C.D. (2019). Model systems for regeneration: *Hydra*. Dev..

[27] Kier W.M. (2012). The diversity of hydrostatic skeletons. J. Exp. Biol..

[28] Han S., Taralova E., Dupre C., Yuste R. (2018). Comprehensive machine learning analysis of *Hydra* behavior reveals a stable basal behavioral repertoire. Elife.

[29] Carter J.A., Hyland C., Steele R.E., Collins E.-M.S. (2016). Dynamics of mouth opening in *Hydra*. Biophys. J..

[30] Campbell R.D. (1987). Structure of the mouth of *Hydra* spp. a breach in the epithelium that disappears when it closes. Cell Tissue Res..

[31] Iachetta R., Ambrosone A., Klimovich A., Wittlieb J., Onorato G., Candeo A., D’andrea C., Intartaglia D., Scotti N., Blasio M. (2018). Real time dynamics of *β*-catenin expression during *Hydra* development, regeneration and wnt signalling activation. Int. J. Dev. Biol..

[32] Maroudas-Sacks Y., Garion L., Shani-Zerbib L., Livshits A., Braun E., Keren K. (2021). Topological defects in the nematic order of actin fibres as organization centers of *Hydra* morphogenesis. Nat. Phys..

[33] Badhiwala K.N., Gonzales D.L., Vercosa D.G., Avants B.W., Robinson J.T. (2018). Microfluidics for electrophysiology, imaging, and behavioral analysis of *Hydra*. Lab A Chip.

[34] Badhiwala K.N., Primack A.S., Juliano C.E., Robinson J.T. (2021). Multiple neuronal networks coordinate *Hydra* mechanosensory behavior. Elife.

[35] Edelstein A.D., Tsuchida M.A., Amodaj N., Pinkard H., Vale R.D., Stuurman N. (2014). Advanced methods of microscope control using *µ*manager software. J. Biol. Methods.

[36] Guan Z., Lee J., Jiang H., Dong S., Jen N., Hsiai T., Ho C.-M., Fei P. (2016). Compact plane illumination plugin device to enable light sheet fluorescence imaging of multi-cellular organisms on an inverted wide-field microscope. Biomed. Opt. Express.

[37] Steele R.E., Updegrove M.D., Kirolos S.A., Mowery L., Martinez D.E., Bryant P.J. (2019). Reproductive bet-hedging and existence in vernal pools as components of *Hydra* life history. Biol. Bull..

[38] Trembley A. (1744). Mémoires Pour Servir à L’histoire D’un Genre de Ploypes D’eau Douce, à bras en Forme de Cornes.

[39] Wilson E.B. (1891). The heliotropism of *Hydra*. Am. Nat..

[40] Haug G. (1933). Die lichtreaktionen der hydren. Z. Für Vgl. Physiol..

[41] Singer R.H., Rushforth N.B., Burnett A.L. (1963). The photodynamic action of light on *Hydra*. J. Exp. Zool..

[42] Nath T., Mathis A., Chen A.C., Patel A., Bethge M., Mathis M.W. (2019). Using deeplabcut for 3d markerless pose estimation across species and behaviors. Nat. Protoc..

[43] Gilpin W., Prakash V.N., Prakash M. (2017). Flowtrace: Simple visualization of coherent structures in biological fluid flows. J. Exp. Biol..

[44] Gilpin W., Prakash V.N., Prakash M. (2017). Dynamic vortex arrays created by starfish larvae. Phys. Rev. Fluids.

[45] Rodrigues M., Leclère P., Flammang P., Hess M.W., Salvenmoser W., Hobmayer B., Ladurner P. (2016). The cellular basis of bioadhesion of the freshwater polyp *Hydra*. BMC Zool..

[46] Khetan N., Maheshwari S., Athale C.A.A. (2019). Quantitative detachment mechanics of *Hydra* from substrates. Proc. Indian Natn. Sci. Acad..

[47] Shen Y., Siryaporn A., Lecuyer S., Gitai Z., Stone H.A. (2012). Flow directs surface-attached bacteria to twitch upstream. Biophys. J..

[48] Kim A.A., Nekimken A.L., Fechner S., O’Brien L.E., Pruitt B.L. (2018). Microfluidics for mechanobiology of model organisms. Methods in Cell Biology.

[49] Hwang H., Lu H. (2013). Microfluidic tools for developmental studies of small model organisms–nematodes, fruit flies, and zebrafish. Biotechnol. J..

[50] Krishnamurthy D., Li H., Rey F.B., Cambournac P., Larson A.G., Li E., Prakash M. (2020). Scale-free vertical tracking microscopy. Nat. Methods.

[51] Hol F.J.H., Lambrechts L., Prakash M. (2020). Biteoscope, an open platform to study mosquito biting behavior. Elife.

[52] Kumar S., Hol F.J.H., Pujhari S., Ellington C., Narayanan H.V., Li H., Rasgon J.L., Prakash M. (2021). A microfluidic platform for highly parallel bite by bite profiling of mosquito-borne pathogen transmission. Nat. Commun..

