# Supplementary material for: SPIM-Flow: An Integrated Light Sheet and Microfluidics Platform for Hydrodynamic Studies of Hydra
Source: Biology (Basel). 2023 Jan 11;12(1):116. doi: 10.3390/biology12010116 (PMC9856110; doi:10.3390/biology12010116)
Supplement: Supplementary file 1 [file biology-12-00116-s001.zip › Supplementy.pdf]

Supplemental Section—Analysis of Light Sheet Properties

The Rayleigh criterion defines the optical resolution, r, as:

$$r = 0.61\frac{\lambda}{NA}, \quad \text{(S1)}$$

where λ is the wavelength and NA is the numerical aperture of the lens. For small aperture lenses, NA can be approximated by:

$$NA = n\frac{D}{2f}, \quad \text{(S2)}$$

where D is the lens diameter, f the lens focal length, and n is the refractive index of the surrounding medium, which, in our case, was air (n = 1). Combining the two equations yields:

$$r = 1.22\frac{\lambda f}{D}. \quad \text{(S3)}$$

With an excitation wavelength of 488 nm, a beam diameter of 1 mm, and a cylindrical lens with a focal length of 12.7 mm, the light sheet minimum thickness was 7.6 µm.

List of Supplemental Videos

Video S1: *Hydra* feeding behavior (*Artemia franciscana* nauplius or brine shrimp)
Video S2: *Hydra* tentacle sway behavior
Video S3: *Hydra* response to flow—volumetric flow rate of 2 mL/hr (flow is from top to bottom)
Video S4: *Hydra* motion tracking and pseudo-skeleton generation via DeepLabCut (response to flow), with a volumetric flow rate of 2 mL/hr
Video S5: Hydrodynamic pathline visualizations and sample visualizations (original videos and FlowTrace are provided)

Please note, for all recordings, wavelengths of 488 nm were used.

Supplemental Figures

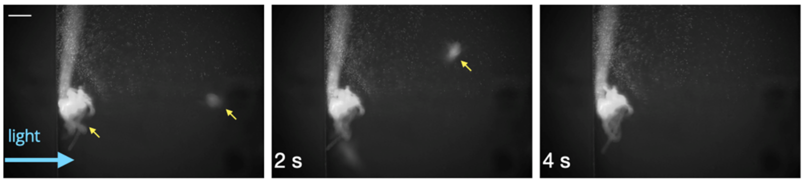

Figure S1. *Hydra*'s feeding behavior as an indicator of health. The *Hydra* specimen previously captured prey. The arrows indicate tentacles holding an *Artemia franciscana* nauplius (brine shrimp). The scale bar is 0.5 mm (wavelengths of 488 nm were used).

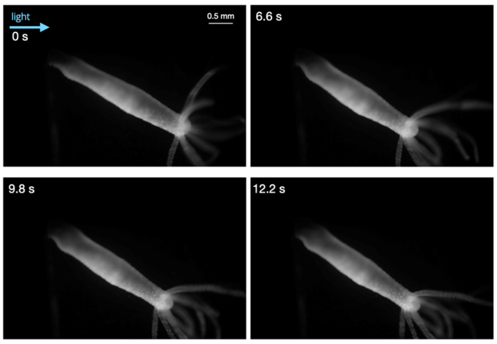

Figure S2. *Hydra*'s baseline behavior in static conditions as an indicator of overall health. The *Hydra* specimen remains in an elongated state as an indicator of overall health. GFP-positive neurons can be visualized depending on the orientation and state of the *Hydra* specimen.

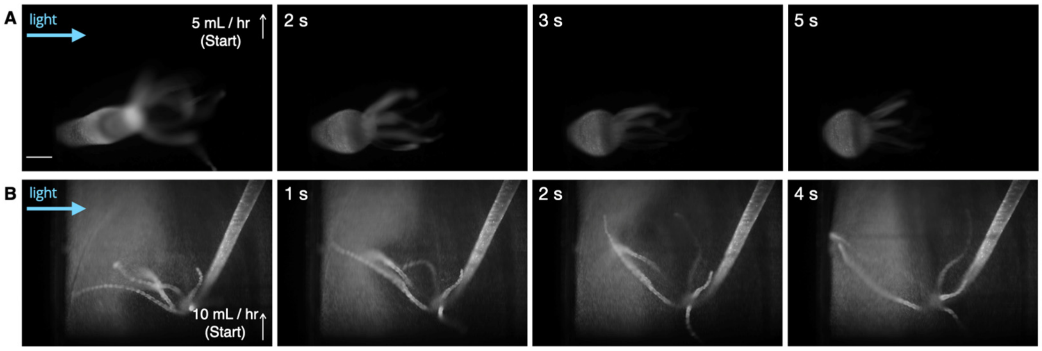

Figure S3. Responses to the initiation of flow. (A) A *Hydra* specimen contracting in response to the flow initiation (flow rate of 5 mL/hr). The scale bar is 0.5 mm. (B) A *Hydra* specimen responding to the flow initiation by exhibiting tentacle-swaying behavior (flow rate of 10 mL/hr). Additional experiments are required to investigate the dynamic response of *Hydra* to various mechanical stimuli.

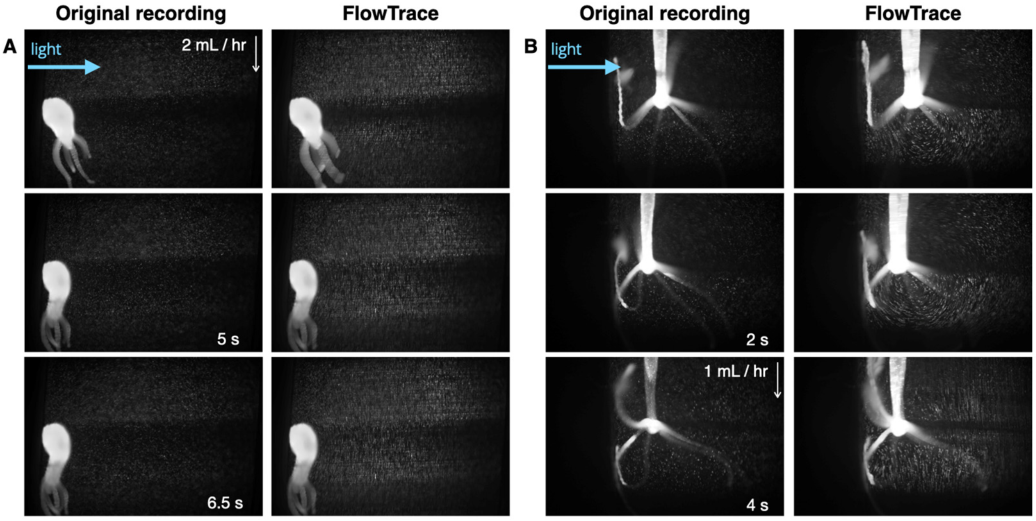

Figure S4. Hydrodynamic visualization via FlowTrace. (A) Original recordings and FlowTrace analysis to better track the bead movement due to the external flow (2 mL/hr). (B) Original recording and FlowTrace analysis. An external flow was initiated (4 s flow, 1 mL/hr). FlowTrace provided a rapid and computationally inexpensive approach for producing path line visualizations from the SPIM-Flow analysis.

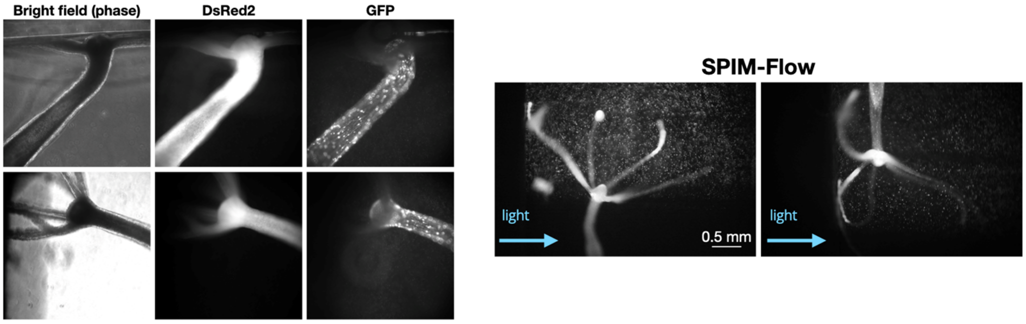

Figure S5. Imaging *Hydra* (N = 2) via a fluorescent microscope and SPIM-Flow (N = 2). We used a commercial fluorescent microscope (Nikon Eclipse Ti-E microscope 10X objective) with a Sola light engine (Lumencor, Beaverton, OR) and 474/27 nm and 575/25 nm filters for excitation and 525/45 nm and 641/75 nm filters for emission for the GFP and DsRed2 visualizations. More anatomical features could be visualized with the microscope. However, SPIM-Flow provided an inexpensive and simple alternative for hydrodynamic and particle tracking studies (excitation 488 nm and 500 nm long pass). Moreover, simultaneous visualizations of the animal and the beads with a standard microscope would require additional optimization.
